# Trends in hepatitis E virus incidence and seroprevalence in the Netherlands, 1988 to 2025

**DOI:** 10.2807/1560-7917.ES.2026.31.43.2600103

**Published:** 2026-10-29

**Authors:** Franke A Quee, Boris M Hogema, Katja van den Hurk, Ed Slot, Hans L Zaaijer

**Affiliations:** 1 Blood-borne Infections, Department of Research, Sanquin Blood Supply Foundation, Amsterdam, the Netherlands; 2 Donor Health, Department of Research, Sanquin Blood Supply Foundation, Amsterdam, the Netherlands; 3 Department of VMS, Pursang Diagnostic Services, Amsterdam, the Netherlands; 4 Department of Public and Occupational Health and the Amsterdam Public Health Research Institute, Amsterdam UMC, Amsterdam, the Netherlands; 5 Department of Medical Affairs, Sanquin Blood Supply Foundation, Amsterdam, the Netherlands

**Keywords:** Hepatitis E Virus, serosurveillance, blood donors, blood donation, screening

## Abstract

**BACKGROUND:**

Hepatitis E virus (HEV) can cause serious liver disease, especially in immunosuppressed patients. In 2013, the Netherlands started HEV-RNA screening of donations for production of solvent detergent (S/D)-treated plasma. Since 2017, all Dutch blood donations are screened.

**AIM:**

To study trends in HEV infection, we analysed the prevalence of HEV antibodies in Dutch donors since 1988, and HEV-RNA screening results since 2013.

**METHODS:**

Prevalence of anti-HEV IgG antibodies was determined in donor samples from 2023, and compared to our previous serosurveillance studies, performed on donor samples collected in 1988, 2000 and 2011. We collected HEV-RNA screening results of S/D plasma donations since 2013, and of all blood donations since 2017.

**RESULTS:**

In 2023, 24.3% of donors tested positive for anti-HEV antibodies. Seroprevalence increased with age. An age-cohort effect contributed to an overall decline in seroprevalence compared with 1988, reflecting historically higher infection pressure. Among donors born after 1950, seroprevalence rose in samples collected after 2000. HEV-RNA screening results initially revealed a high incidence that declined sharply after 2015, followed by an upward trend beginning in 2020. HEV-RNA positivity was more common in male and older donors.

**CONCLUSION:**

The data indicate substantial historical fluctuations of HEV infection pressure. High seroprevalence in the late 20th century suggests widespread past exposure, followed by a prolonged period of reduced infection rates. Around 2000, HEV appears to have re-emerged, with increasing seroprevalence among younger donors. Screening has revealed marked year-to-year variability in incidence, underscoring dynamic patterns of HEV circulation.

**Key public health message**

**What did you want to address in this study and why?**

Hepatitis E virus (HEV) infection can cause severe chronic liver disease, especially in immunosuppressed patients. Blood donors can be infected without showing symptoms and thus blood component testing is needed to prevent transmission of HEV. To evaluate the need for donor screening in the Netherlands, we wanted to understand how many donors had a past HEV infection and how many had an active HEV infection when donating.

**What have we learnt from this study?**

Our data from 2023 show that approximately a quarter of the Dutch donor population has had an HEV infection at some point. After years of a decreasing trend in seroprevalence, a plateau has possibly been reached. HEV-RNA screening shows that since 2014, the number of people with an active HEV infection rapidly decreased but from 2020, an upward trend can be observed.

**What are the implications of your findings for public health?**

Our data underscore that HEV is still circulating in the Netherlands. As the number of donors who have had an HEV infection seems to have reached a plateau and the number of donors with an active HEV infection does not decrease further, these dynamics do not support cessation of HEV-RNA donor screening.

## Introduction

Hepatitis E virus (HEV) is a non-enveloped RNA virus that can cause severe liver disease. Of the four major genotypes (gt1–4) that can infect humans, transmission routes and prominent endemic areas differ. Genotypes 1 and 2 are mainly transmitted from human to human via contaminated water and are endemic in Asia, Latin-America, and Africa [1,2]. Genotypes 3 and 4 are zoonotic and mainly transmitted via contaminated food and water. Genotype 3 is found in pigs worldwide, while genotype 4 is mostly found in pigs in Eastern Asia [3]. In Europe, most infections are caused by HEV-gt3. Pork consumption in the Netherlands is high, and the country is one of the biggest meat exporters in the European Union (EU), rearing ca 11 million pigs each year. Hence, monitoring and understanding the dynamics of this zoonotic infection is important [4,5].

HEV-gt3 is transfusion transmissible and poses a threat to blood safety. Infection can cause serious chronic disease, especially in immunosuppressed patients including those with haematological conditions and organ transplant recipients. As healthy people often display mild or no symptoms of illness when infected with HEV-gt3, blood donors may donate while being asymptomatically infected or before developing symptoms. Recipients are then at risk for acquiring infection and developing disease if transfused with HEV-infected blood components [6].

In the period 2005–2015, an increasing number of HEV-gt3 infections and related hospitalisations were reported in European countries [7,8]. To investigate the situation in the Netherlands, where HEV is not a notifiable disease, a serosurveillance study was conducted in 2011, which found an HEV seroprevalence of 27% in Dutch donors [9]. RNA screening for HEV was started in 2013 for all Dutch donations collected for the production of solvent detergent (S/D)-treated plasma. Screening of this type of plasma is mandatory, because the combination of sample pooling and insufficient virus inactivation by the S/D treatment could result in HEV infection of large numbers of patients from one strongly positive batch. Because a high incidence of HEV infection was observed in this screening, with 1 in 762 donations testing HEV-positive between January 2013 and December 2014 [10], HEV-RNA screening of all Dutch donations was started in May 2017 of all short-shelf-life blood products, such as erythrocyte and thrombocyte concentrates. The primary rationale was to protect transfused organ transplant recipients and other immunocompromised individuals for whom HEV infection carries a significant risk of chronicity and severe outcomes. In 2018, the Dutch Health Council advised the continuation of HEV-RNA donor screening, as long as measures to protect donors against HEV infection are not implemented and evaluated [11].

In this study, we determined the prevalence of anti-HEV antibodies in Dutch blood donors in 2023 and compared with our earlier findings in donor samples from 1988, 2000 and 2011, using the same antibody assay [12]. Additionally, we report data from 13 years of HEV-RNA screening, thus providing a complete context for policymaking regarding transfusion safety and the screening of donors for HEV in the Netherlands.

## Methods

### Study setting and population

Sanquin is the organisation responsible for the collection and distribution of blood components in the Netherlands. Over 400,000 non-remunerated voluntary donors make more than 750,000 donations per year at one of the fixed or mobile blood collection sites throughout the Netherlands [13]. Donors must be aged between 18 and 79 years, be in good health without risk for transfusion-transmissible infections, and pass the pre-donation health check. In 2018, the maximum age for donation was changed from 64 to 79 years. As a consequence, the age distribution in the subsequent serological studies is different. In the Netherlands, HEV infection is not notifiable and surveillance of the virus is limited.

### Hepatitis E virus serosurveillance study

In a cross-sectional study, we performed anti-HEV antibody measurements in over 2,000 blood donations collected between 4 and 7 September 2023. Samples were collected from donors who provided written informed consent for the use of their information and leftover material for research purposes, as part of routine blood collection procedures. We aimed to select samples that were equally distributed across 10-year age groups and sex, with around 200 donors in each age group. Samples were collected from across the country to ensure even geographical representation. 

Plasma samples were tested using the Wantai HEV-IgG ELISA (Beijing Wantai Biological Pharmacy Enterprise Co., Ltd) according to the manufacturer's instructions. Samples with a signal-to-cutoff of > 1.1 were considered positive. Samples with a signal-to-cutoff > 0.5 were retested for confirmation. Discrepant samples were resolved with a second re-test, resulting in re-classification of the original result for only one sample. A panel of samples collected from our previous serological study in 2011 was re-tested using the current kit lot and showed very similar reactivity and complete qualitative agreement (data not shown). 

In addition to the anti-HEV antibody results, data on age, sex and region of donors were available in the donor database.

### Hepatitis E virus RNA screening

HEV-RNA screening was introduced in the Netherlands in January 2013 for S/D plasma donations. Screening was performed in pools of 96 samples with an in-house test (95% LOD = 38.4 IU/mL)[9]. From July 2017, all short-shelf-life products, such as erythrocytes and thrombocytes, were screened using the Roche Cobas HEV test in pools of 24 (Roche Diagnostics; 95% LOD = 18.6 IU/mL). For the present analysis, we included data on testing of S/D plasma collected between January 2013 and June 2017 and data on short-shelved products collected between July 2017 and July 2025. 

Although screening of S/D plasma continued in pools of 96 samples after July 2017 using the in-house test, these data are not included because of the smaller number of samples. After July 2017, the trends in the percentage of positive samples were highly similar. The incidence in S/D plasma was overall slightly lower (most likely because of the less sensitive test) but the difference in HEV positive samples between the two testing approaches per calendar year was never significant (data not shown).

### Statistics

Seropositivity rates are reported as proportions. Differences between age groups and birth cohorts were tested using a chi-square test. Regions were categorised into north (provinces of Groningen, Flevoland, Overijssel, Drenthe, Friesland), mid-east (Gelderland), mid-west (Utrecht, North Holland), south-east (North Brabant, Limburg), and south-west (South Holland, Zeeland). Sero-surveillance results were compared with previously collected data from Hogema et al. [12], where HEV seroprevalence data were obtained by testing stored samples from donations made in 1988, 2000 and 2011 with the Wantai IgG assay. 

HEV-RNA screening results are reported per month (until 2017) or week (starting 2017) as positive samples per 1,000 donations. Donations with an unknow donor identification number or donation date were removed. To visualise trends, the moving 5-month average is shown. The 5-month average was calculated as the mean of the observation for a given month/week, the two preceding two months/weeks, and the two subsequent months/weeks. For donations screened after July 2017, biological sex and age is reported. Age is categorised in 10-year age groups, with the exemption of the youngest (18–30 years) and oldest (71–76 years) age groups. 

## Results

### Hepatitis E virus serosurveillance

In the cross-sectional study conducted in 2023, 2,181 blood donations were included of which 998 were from women and 1,193 from men (Table 1). The overall HEV seroprevalence in 2023 was 24.3% (531/2,181). Samples with an OD/CO > 0.5 (n = 918) were re-tested, showing good alignment with the initial results. The comparison between the initial test result and the re-test is provided in Supplementary Figure S1. Seroprevalence was highest in the south-east region of the Netherlands, for both men and women. Seroprevalence was stable at 20% in the age groups between 31 and 60 years and increased to 39% in donors older than 70 years (Figure 1A, Table 1).

**Table 1:** Hepatitis E virus seroprevalence in blood donors per age group, birth cohort and geographic region in a cross-sectional serosurveillance study, the Netherlands, 2023 (n = 2,181)

Characteristics	Women (n = 988)			Men (n = 1,193)			Total (n = 2,181)			p value	X^2^ test^ a^
	Anti-HEV IgG	Donors	%	Anti-HEV IgG	Donors	%	Anti-HEV IgG	Donors	%		
Total	217	988	22.0	314	1,193	26.3	531	2,181	24.3	0.05	5.33
Age group (years)											
18–30	27	150	18.0	26	197	13.2	53	347	15.3	ns	
31–40	29	149	19.5	46	208	22.1	75	357	21.0	ns	
41–50	33	192	17.2	48	207	23.2	81	399	20.3	ns	
51–60	33	175	18.9	40	181	22.1	73	356	20.5	ns	
61–70	48	197	24.4	71	188	37.8	119	385	30.9	0.01	7.47
71–76	47	125	37.6	83	212	39.2	130	337	38.6	ns	
Birth cohort											
1941–1950	31	76	40.8	45	128	35.2	76	204	37.3	ns	
1951–1960	57	206	27.7	93	240	38.8	150	446	33.6	0.05	5.61
1961–1970	29	177	16.4	56	185	30.3	85	362	23.5	0.01	8.95
1971–1980	40	198	20.2	40	201	19.9	80	399	20.1	ns	
1981–1990	29	163	17.8	48	211	22.7	77	374	20.6	ns	
1991–2000	25	123	20.3	29	174	16.7	54	297	18.2	ns	
2001–2010	6	45	13.3	3	54	5.6	9	99	9.1	ns	
Geographic region											
Mid-east	24	114	21.1	38	155	24.5	62	269	23.0	ns	
Mid-west	44	199	22.1	56	221	25.3	100	420	23.8	ns	
North	47	227	20.7	77	252	30.6	124	479	25.9	0.05	5.54
South-east	68	236	28.8	86	280	30.7	154	516	29.8	ns	
South-west	34	212	16.0	57	285	20.0	91	497	18.3	ns	

HEV: hepatitis E virus; ns: not significant.

^a^ Differences between men and women were tested using the chi-square test.

Percentage of anti-HEV IgG-positive samples are presented by row.

**Figure 1:**
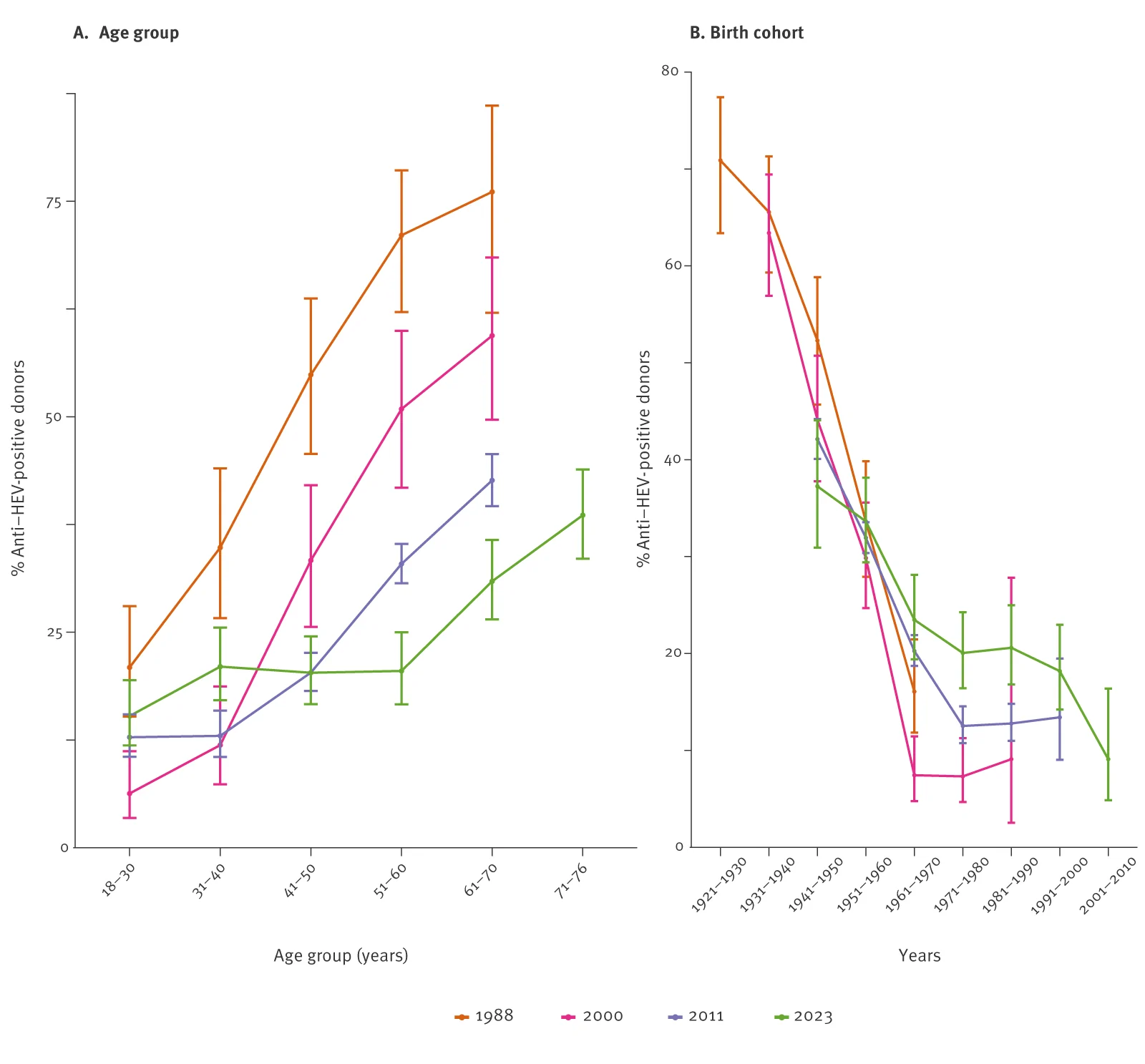
Hepatitis E virus seroprevalence in blood donors by (A) age group and by (B) birth cohort and year of donation, the Netherlands.

HEV: hepatitis E virus.

Hepatitis E virus seroprevalence was measured using the Wantai HEV IgG assay. Error bars represent 95% confidence intervals. The data from our previous seroprevalence studies (1988, 2000, and 2011), was derived from Hogema et al. [12].

Comparing these findings with our previous studies, the age-adjusted seroprevalence for donors aged 18 to 64 declined from 47% in 1988 to 27% in 2000 and to 21% in 2011 [12]. In 2023, the seroprevalence in this age group was 21%, possibly reaching a plateau. Hepatitis E virus seroprevalence decreased in donors aged > 50 years over time, from 71% to 21% in donors aged 51–60 (p < 0.001, X^2^ = 97.9) and 76% to 31% in donors aged 61–70 (p < 0.001, X^2^ = 34.6) when comparing 1988 with 2023 [12] (Table 1). In contrast, after 1988, seroprevalence in younger donors (< 40 years) increased over time, from 6% to 15% in donors aged 18–30 (p < 0.01, X^2^ = 7.2) and 12% to 21% in donors aged 31–40 (p < 0.05, X^2^ = 4.5) when comparing 2000 with 2023. Figure 1B presents the seroprevalences by birth cohort, showing that the seroprevalence decreased as the year of birth was more recent.

### Hepatitis E virus RNA screening

From January 2013 to June 2017, 124,275 S/D plasma donations were screened, and 97 donations were HEV-RNA positive (0.78/1,000 donations). Since July 2017, screening of all short-shelf-life donations has been implemented, with 3,304,302 donations screened up to August 2025. We removed 449 (0.01%) donations with an unknown donor identification number or donation date, leaving a total of 3,303,853 donations in the period 2017–2025. From July 2017 to July 2025, 894 donations were HEV-RNA positive (0.27/1,000 donations). HEV incidence peaked in 2014 and 2015, with over 1 in 1,000 donations testing positive for HEV-RNA (Table 2, Figure 2). Between 2014 and 2020 the HEV incidence strongly declined from 0.12% to 0.02% positive donations (p < 0.01, X^2^ = 79.4). After 2022, the infection rate showed a slow upward trend (2022 vs 2024, p < 0.01, X^2^ = 6.9).

**Table 2:** Hepatitis E virus RNA screening per year in blood donations, the Netherlands, 2013–2025 (n = 3,428,225)

Screening year	HEV-RNA-negative samples (n)	HEV-RNA-positive samples (n)	HEV-positive samples per 1,000 donations
S/D plasma screening			
2013	35,214	18	0.51
2014	24,260	28	1.15
2015	22,316	25	1.12
2016	30,557	17	0.56
2017 (until June)	11,928	9	0.75
**Total**	**124,275**	**97**	**0.78**
Screening all donations for short-shelf-life products^a^			
2017 (from July)	203,804	104	0.51
2018	422,989	154	0.36
2019	424,201	105	0.25
2020	429,398	86	0.20
2021	420,063	97	0.23
2022	406,273	81	0.20
2023	396,148	97	0.24
2024	375,166	106	0.28
2025 (until July)	224,917	64	0.29
**Total**	**3,302,959**	**894**	**0.27**

HEV: hepatitis E virus; S/D: solvent detergent-treated plasma.

^a^ In 2017, screening for short-shelf-life products was implemented due to high HEV incidence in the Netherlands.

**Figure 2:**
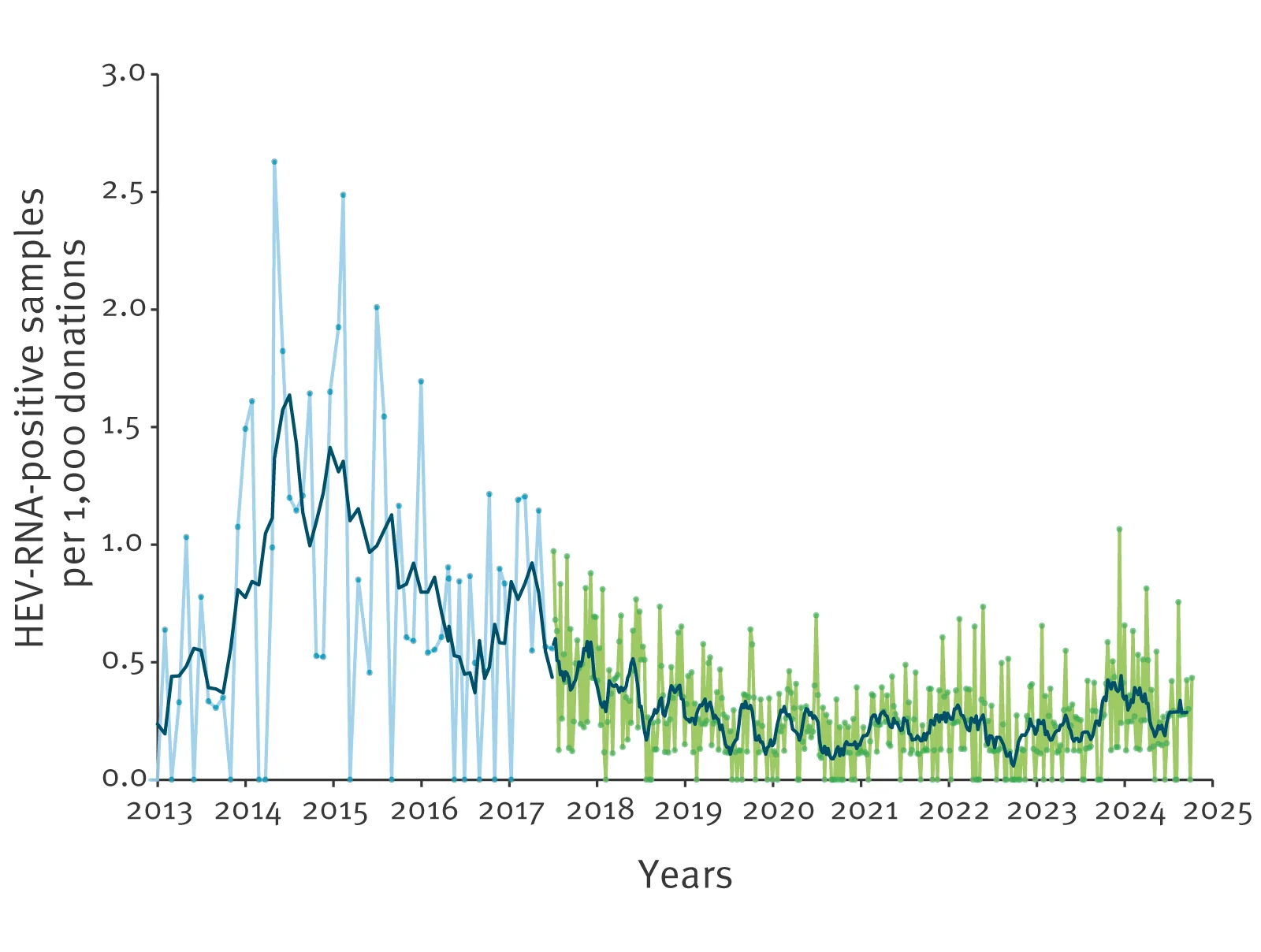
Monthly hepatitis E virus RNA screening yields for S/D plasma, January 2013–June 2017 (n = 124,275) and weekly HEV-RNA screening of all blood donations, July 2017–July 2025 (n = 3,303,853)

HEV: hepatitis E virus; S/D: solvent detergent-treated plasma.

The blue line shows the HEV-RNA screening results for S/D plasma, the blue dots represent the number of HEV-RNA-positive samples/1,000 donations per month. The green line shows the HEV-RNA screening results of the screening of all short-shelf-life donations, the green dots represent the number of HEV-RNA-positive samples/1,000 donations per week. The dark blue line shows the moving 5-month average.

When stratified by age, the age group 18–30 years and, to a lesser extent, the group of 31–40 years show lower infection rates compared with the older age groups (Figure 3A). The number of HEV-RNA-positive donations in the age group 71–77 fluctuates over time, probably caused by the small size of the group. The small group size can be explained by the fact that before April 2018, donors aged 70 years or older were not allowed to donate. The number of HEV-RNA-positive donations stratified by year and age group or sex are provided in Supplementary Tables S2 and S3. The HEV infection rates in men and women show the same trend over time, with significantly higher infection rates in men as compared with women in 2018, 2020 and 2021 (p < 0.05; Figure 3B).

**Figure 3:**
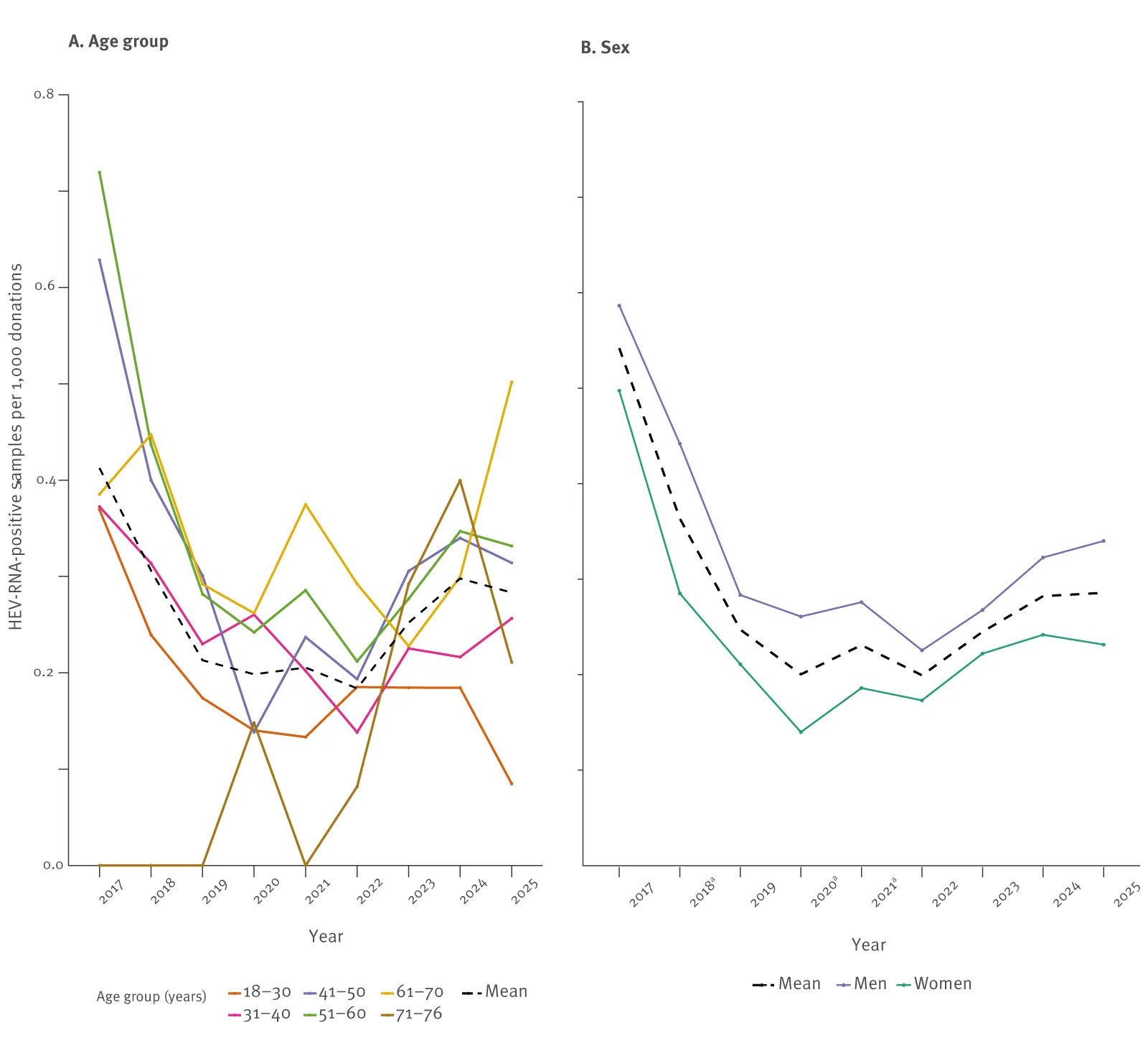
Hepatitis E virus RNA-positive samples per 1,000 donations over time by (A) age group and (B) sex, the Netherlands, 2017–2025 (n = 3,302,954)

HEV: hepatitis E virus.

^a^ Men and women significantly differ in the indicated year (p < 0.05).

HEV-RNA yields per year are shown stratified by age group and sex, the dashed line represents the mean.

## Discussion

The testing for HEV antibodies suggests that in 2023, approximately a quarter of the Dutch donor population experienced HEV infection sometime during life. Our study shows that, after years of a decreasing trend in seroprevalence, possibly a plateau has been reached. The data collected in 2023 show a positive correlation between seroprevalence and age, similar to the previous studies [12]. Interestingly, seroprevalence in younger donors increased in 2023 as compared with 2000 and 2011. The HEV-RNA screening shows that since 2014, HEV incidence rapidly decreased. However, since 2020, an upward trend can be observed.

Comparison of the seroprevalence studies at four timepoints between 1988 and 2023 shows that the HEV seroprevalence strongly decreased in time. This can be explained by an age-cohort effect in donors born before 1940: based on the maximum donation age, the donors with the highest seroprevalence are no longer eligible to donate and are not monitored in later studies. Based on the prevalence of more than 50% and on an age-dependent slope in donors born before 1950 in the oldest sample sets, it can be deduced that the incidence in the decades before the 1988 study surpassed the incidence of 1.1% per year as observed between 2009 and 2011 by Slot et al. [14]. After decades with (very) low infection rates, HEV seems to have re-appeared around the year 2000, causing an increasing seroprevalence in non-immune younger donors (< 41 years). The seroprevalence studies suggest that the prevalence among older donors (born before 1961), defined by the birth cohorts, was rather stable between 1988 and 2023 and is not significantly increasing with time as in younger donors. 

In contrast, the results from donor HEV-RNA screening show that active infection is more frequently observed among older donors. This apparent discrepancy may be explained by seroreversion of donors long after their infection. This would logically occur more frequently in older donors (since antibodies may be detectable for decades) and result in an underestimation of the prevalence of past HEV infection in this group. Seroreversion was observed in 22% of HEV-seropositive participants over a period of 12 years in a German study, but this was measured using an immunoblot that is somewhat less sensitive than the Wantai IgG assay [15]. To our knowledge, the only large study addressing seroreversion using the Wantai HEV IgG assay showed seroreversion in 6 of 172 anti-HEV-positive individuals in samples taken on average 3 years apart [16]. Our own sero-surveys did not use linked donors. To compensate for this, we previously tested samples from 23 donors who tested positive in 1988 and for whom samples from 2011 were available. In that small study, five donors seroreverted, all with relatively low S/CO values in 1988 [12]. It would be useful to repeat this study at a larger scale to obtain accurate estimates of the frequency of seroreversion and the resulting underestimation of past HEV incidence in serological studies.

Our data show modest differences in prevalence between regions of the country. The highest prevalence was noted in the south-east, which is also the area with the highest pig density in the Netherlands. Consumption of (undercooked) pork products, such as liver sausages is a major risk factor for HEV infection [17]. Consumption of pork in the EU and the Netherlands has been relatively stable over the last 20–30 years [18,19]. We are not aware of differences in culinary habits and meat consumption in different areas of the Netherlands. In the period between 2000 and 2023, the number of pig farms in the Netherlands decreased, from approximately 13,000 farms in 2000, to approximately 2,500 in 2023 [20]. This decline was accompanied by a reduction in meat pigs in the Netherlands, but an increase from 500 to 1,900 pigs on average per farm. A higher concentration of pigs could lead to easier spread of HEV between animals. Studies showed that implementation of biosecurity measures on meat pig farms could reduce the number of HEV-infected pigs and therefore protect pork product consumers against HEV [21,22]. Since HEV infection does not cause notable disease in pigs, monitoring of pigs has limited priority, and it seems unlikely that voluntary preventive measures will be implemented. 

Recent European studies on HEV-RNA incidence show a wide range of results. Finland reported a relatively low incidence of 17 HEV-RNA-positive samples per 100,000 donations between 2020 and 2021 using individual NAT testing [23]. In Switzerland, an incidence of 20.7 positive samples per 100,000 donations, tested in mini-pools of 12–24 donations, was reported over a 5-year period (2018–23) [24]. In Russia, an incidence of around 25 positives per 100,000 donations, tested in mini-pools of six samples, was reported in 2022–2023 [25]. Germany reported  ca 68 HEV-positive samples per 100,000 donations, tested in pools of 96, between 2015 and 2022 [26]. France reported the highest incidence, 100 positives per 100,000 donations in the period between 2014 and 2021, tested in mini-pools of six [27]. Clearly, the sample pool size will affect the percentage of positive donations observed. Based on the viral load distribution, we estimate that screening in pools of 24 using the Roche test results in non-detection of ca 50% of the positive donations [28]. In our study, we report an average incidence of 27 positive samples per 100,000 donations, but with large fluctuations in time of 115 to 20 positive samples per 100,000 donations. Such large fluctuations in time, based on surveillance using the same test and pool size have not been reported before in other countries. The cause of these fluctuations is yet unknown.

The decision to perform HEV screening of blood products is challenging. Apart from S/D-treated plasma, which must test negative for HEV-RNA after pooling to comply with the European Pharmacopoeia, there are no clear regulations. While many countries decided to implement screening since 2012, a substantial proportion of European countries decided to not implement screening, and there appears to be a very limited correlation between the decision to screen and the estimated incidence [29]. The decision to implement screening in the Netherlands was based on the very high prevalence noted in the first years of screening S/D-treated plasma. It was estimated that screening would prevent > 150 HEV transmissions per year, of which 4 to 5 infections would become chronic and potentially life threatening in organ transplant recipients [28]. The fivefold reduction in HEV incidence observed among Dutch donors between 2015 and 2018 reduced the yield of the HEV screening, and therefore the cost effectiveness of the measure. Nevertheless, HEV screening prevents more virus transmissions than other screening tests do and cessation of screening would also end the only systematic way in which HEV infections are monitored in the Netherlands [30].

This recent HEV seroprevalence study in the Netherlands provides information on HEV-RNA screening of humans on a large scale. The unbiased approach to sampling is a strength, as the sample was constructed without taking risk of exposure into account. However, the limited number of HEV-RNA-positive samples and the fluctuations of the incidence over time may have shielded smaller seasonal variations or small differences between age groups and sex. Blood donor data are prone to be biased due to the healthy donor effect, a selection bias caused by the health criteria for blood donation. Donors who feel ill, potentially related to an HEV infection, are not allowed to donate and are therefore not captured by screening. This could lead to an underestimation of the HEV incidence. However, since HEV infection is most often asymptomatic, we assume that this bias is negligible. 

## Conclusion

In the Netherlands, HEV incidence decreased since 2014, but it shows a slow upward trend since 2020. Overall, the HEV seroprevalence in Dutch donors possibly has reached a plateau, but we found an increase in seroprevalence in younger donors. This could be due to recent re-appearance of HEV, or increased exposure of younger individuals. The seroprevalence in older donors decreased. These dynamics and uncertainties do not support the cessation of HEV-RNA donor screening.

## Data Availability

All data presented in the manuscript or supplementary material are available from the corresponding author upon reasonable request.

## Supplementary Data

174000 Supplement
