# Supplementary material for: Trends in hepatitis E virus incidence and seroprevalence in the Netherlands, 1988 to 2025
Source: Euro Surveill. 2026 Oct 29;31(43):2600103. doi: 10.2807/1560-7917.ES.2026.31.43.2600103 (PMC13722930; doi:10.2807/1560-7917.ES.2026.31.43.2600103)
Supplement: Supplementary file 1 [file 26-00103_QUEE_Supplement.pdf]

This supplementary material is hosted by Eurosurveillance as supporting information alongside the article “Trends in hepatitis E virus incidence and seroprevalence in the Netherlands, 1988 to 2025” on behalf of the authors who remain responsible for the accuracy and appropriateness of the content. The same standards for ethics, copyright, attributions and permissions as for the article apply. Eurosurveillance is not responsible for the maintenance of any links or email addresses provided therein.

Supplementary Figure S1. Results of repeat testing of samples with an OD/CO ratio>0.5

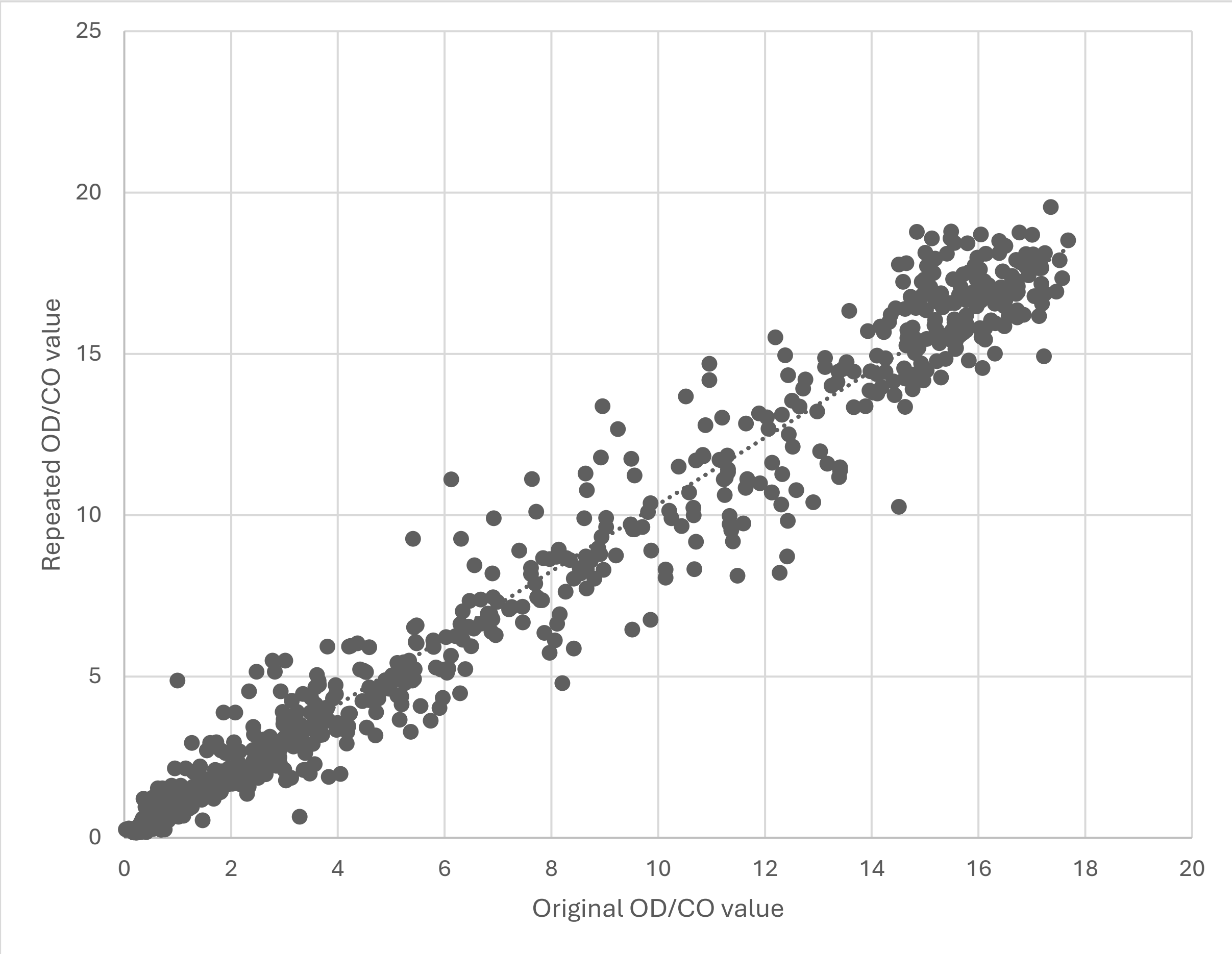

Supplementary Table S2. Number of HEV-RNA positive donations per total donations stratified by year and age group.

**year**	18-30	31-40	41-50	51-60	61-70	71-77	**Total**
2017	18/38945	8/23114	22/33364	37/48307	12/34197	0/0	97/177927
2018	23/95022	17/52493	29/71249	14/98497	34/74011	0/621	144/391893
2019	20/115038	14/60855	22/73103	27/95784	22/75342	0/3859	105/423981
2020	17/121253	17/65291	10/72233	23/95008	18/68735	1/6756	86/429276
2021	15/112493	13/64351	17/71712	27/94523	25/66714	0/10188	97/419981
2022	18/97154	9/965102	14/72356	20/94405	19/64996	1/12222	81/406235
2023	17/92086	15/66581	22/71985	25/90314	14/61496	4/13695	97/396157
2024	15/81314	14/64702	23/67673	30/86456	18/60035	6/15014	106/375194
2025	4/47134	10/38982	13/41382	17/51254	18/35875	2/9479	64/224106

Supplementary Table S3. Number of HEV-RNA positive donations per total donations stratified by year and sex.

Year	Women	Men
**2017**	41/82430	56/95497
**2018**	53/185922	91/205971
**2019**	44/208714	61/215268
**2020**	30/214565	56/214711
**2021**	39/209715	58/210266
**2022**	35/202242	46/203994
**2023**	44/198156	53/198001
**2024**	45/186140	61/189054
**2025**	26/112332	38/111775
